# Comparison of a 4-Day versus 2-Day Low Fiber Diet Regimen in Barium Tagging CT Colonography in Incomplete Colonoscopy Patients

**DOI:** 10.1155/2015/609150

**Published:** 2015-03-22

**Authors:** Kaan Meric, Nuray Bakal, Ebubekir Şenateş, Sibel Aydın, Zeynep Gamze Kılıçoğlu, Fatma Esra Bahadır Ülger, Esin Yencilek, Banu Erkalma Şenateş, Masum Şimşek

**Affiliations:** ^1^Department of Radiology, Haydarpasa Numune Training and Research Hospital, 34668 Istanbul, Turkey; ^2^Department of Radiology, Zeynep Kamil Training and Research Hospital, 34668 Istanbul, Turkey; ^3^Department of Gastroenterology, Göztepe Education and Research Hospital, Istanbul Medeniyet University, 34730 Istanbul, Turkey; ^4^Department of Radiology, Fatih Sultan Mehmet Training and Research Hospital, 34752 Istanbul, Turkey; ^5^Department of Internal Medicine, Cerrahpaşa Medical Faculty, Istanbul University, 34098 Istanbul, Turkey

## Abstract

Our aim was to compare the amount of residual feces, residual fluid, the tagging quality, and patient compliance using 4-day versus 2-day low fiber diet regimen in barium tagging CT colonography in incomplete colonoscopy patients.* Methods.* A total of 101 patients who underwent CT colonography were assigned to 2-day diet group (*n* = 56) and 4-day diet group (*n* = 45). Fecal tagging was achieved with barium sulphate while bisacodyl and sennoside B were used for bowel preparation. Residual solid stool was divided into two groups measuring <6 mm and ≥6 mm. We graded the residual fluid, tagging quality for solid stool, and fluid per bowel segment. We performed a questionnaire to assess patient compliance.* Results.* 604 bowel segments were evaluated. There was no significant difference between 2-day and 4-day diet groups with respect to residual solid stool, residual fluid, tagging quality for stool, and fluid observed in fecal tag CT colonography (*P* > 0.05). The prevalence of moderate discomfort was significantly higher in 4-day group (*P* < 0.001).* Conclusion.* Our study shows that 2-day limited bowel preparation regimen for fecal tag CT colonography is a safe and reasonable technique to evaluate the entire colon, particularly in incomplete conventional colonoscopy patients.

## 1. Introduction

Conventional colonoscopy (CC) is currently the gold standard method for screening colorectal diseases [1, 2]. However, about 6–26% of examinations are incomplete and fail to reach the initial tract of the colon and caecum [3, 4]. The common reasons for incomplete colonoscopy are redundant or tortuous colon, angulation or fixation of colonic loops, marked diverticular disease, adhesions due to prior surgery, obstructing masses and strictures, and spasm or poor colonic preparation [5].

American Gastroenterological Society recognizes computed tomographic colonography (CTC) as the imaging modality of choice in case of incomplete CC [6]. CTC is a noninvasive imaging technique that has the advantages of rapid data acquisition, minimal patient discomfort, lack of need for sedation, and virtually no recovery time [7]. Use of fecal tagging (FT) before CTC in combination with a reduced cathartic cleansing has been proven to be an efficient method for screening the entire colon [8]. In addition, low fiber diet reduces the residual bowel content [9]. In clinical practice, the CTC patients are generally prepared with a low fiber diet two days prior to the examination [8, 10, 11].

Although CTC is generally well tolerated by patients [12, 13], the bowel preparation still remains to be a challenge for patients [13]. We hypothesized that longer (4 days) bowel preparation with low fiber diet and markedly reduced cathartic agents before FT-CTC could have better results in terms of residual solid stool, residual fluid, and tagging quality. For this purpose, we compared the amount of residual solid stool and fluid, the tagging quality, and patient compliance using a 2-day versus 4-day limited bowel preparation regimen for FT-CTC in incomplete CC patients.

## 2. Materials and Methods

### 2.1. Patients

One hundred and one patients (mean age 57 years, range 33–85) who have undergone CTC (90 due to incomplete CC and 11 due to relative contraindication of CC) between January 2011 and January 2013 were enrolled in this study. Fifty patients were female (49.5%) and 51 were male (50.5%). Fifty-six patients were assigned to 2-day diet group and this group was referred to as “2-day” and the remaining 45 patients were assigned to 4-day diet group and this group was referred to as “4-day.” The radiologist who was blinded to patient clinical information has included the patients into one of the above mentioned two groups in a random way.

The indications for CC evaluation were various: change in bowel habits (*n* = 28), abdominal pain (*n* = 25), anemia (*n* = 16), rectal bleeding (*n* = 13), positive fecal occult blood test (*n* = 12), family history of colorectal cancer (*n* = 6), and follow-up after colectomy (*n* = 1). Among those 101 patients, 11 (11%) underwent CTC due to relative contraindication of CC for the following reasons: accompanying cardiovascular and pulmonary disorders (*n* = 5), anticoagulation therapy (*n* = 3), and poor tolerance for CC preparation (*n* = 3). Ninety (89%) of the 101 patients were referred to CTC because CC was incomplete due to poor colonic preparation and presence of residual colonic content (*n* = 48), patient intolerance during CC examination (*n* = 22), sigmoid diverticular disease (*n* = 10), colonic tortuosity (*n* = 8), and adhesions (*n* = 2). CTC was performed within 2 weeks after incomplete colonoscopy (mean interval 12 days). The study was performed according to the World Medical Association Declaration of Helsinki. All patients included in this study have signed informed consent.

### 2.2. Bowel Preparation

For each of the regimens, patients followed a low-residue diet 2 days before the CTC for 2-day group and 4 days before the CTC for 4-day group. During this period, patients were instructed to avoid intake of all fiber-rich food, including fruit, vegetables, whole-grain bread, and whole-grain cereals. The day prior to CTC, these patients were allowed only a liquid diet before the examination. This diet consisted of only clear and opaque liquid foods with a smooth consistency [9].

FT was achieved using 225 mL barium sulphate suspension (E.Z.CAT barium sulphate suspension, Opakim Medical Products, Canada) which was diluted with 500 mL of water or any fruit juice without pulp (2.1% w/v barium sulphate). The suspension was divided into three equal portions. For 2-day diet group, it was given to patients on the 2nd day, 15 minutes after breakfast, lunch, and dinner. For 4-day diet group, it was given on 4th day, 15 minutes after breakfast, lunch, and dinner. Bisacodyl (5 mg) and sennoside B (3 mg) (Bekunis, Abdi Ibrahim, Turkey) with stool softening effects were used for bowel preparation. Both diet groups took 2 pills at 7.00 PM on the night prior to the examination. In the morning before the procedure, no breakfast was allowed. CTC was performed between 8:30 and 9:30 AM.

None of patients were excluded from the study because of complete patient compliance to both dietary regimens.

### 2.3. CTC Technique

All CTC examinations were performed with use of a 64-detector row scanner (Toshiba Aquilion TSX-101A, Japan). Prior to CTC, in an attempt to minimize bowel peristalsis and to reduce possible colonic spasm, a spasmolytic agent (20 mg hyoscine-N-butyl bromide, Buscopan, Eczacibasi, Turkey) was administered intravenously. In the left lateral decubitus position, the colon was gently insufflated with room air by a radiologist using a lubricated foley catheter placed in the rectum until the patient requested that air insufflation be discontinued or distention was believed to be adequate (30–50 bulb compressions). Colonic distention was assessed on the scout view. In the event of insufficient colonic distention, additional insufflation was performed. In the event of good colonic distention, the patient underwent scanning while supine with the tube still in the rectum. The patient was subsequently turned to the prone position. If necessary, additional inflation of the colon was performed. If the patient was not able to lie in prone position, the patient was scanned in the left decubitus position. To avoid concealment of rectal polyps by the rectal tube, the tube was removed at this time.

CT scans were obtained with 64 × 0.5 mm detector collimation at 120 kV and 100 mAs (0.8 pitch and 0.5 sec gantry rotation, 5 mm slice thickness, and 0.5 mm reconstruction interval). To reduce radiation exposure, patients were scanned at a reduced dose of 50 mAs in the prone position. Whole abdomen from the diaphragm to the symphysis pubis was scanned in a single breath hold in an average of 8 seconds. A radiologist recorded any complications associated with CTC.

### 2.4. CTC Image Analysis and Grading of Bowel Preparation

The CT data sets were postprocessed using commercially available software (Aquarius iStation, TeraRecon, CA, USA). Two radiologists (both with experience of over 200 validated CTC cases) who were blinded to the administered diet, retrospectively, reviewed all CTC examinations in consensus. They graded the quality of preparation and success of tagging by using a system adapted from Taylor et al. and Lefere et al. [10, 11]. The colon was evaluated per patient and on a segmental basis. For analysis purposes, colon was divided into six segments: caecum, ascending colon, transverse colon, descending colon, sigmoid colon, and rectum.

We recognised residual solid stool such as pieces of feces mobile with supine and prone positions, tagging with barium without layering; if it has not been tagged it could be recognised as showing presence of air inclusions. Residual solid stool was divided into two groups measuring <6 mm and ≥6 mm (based on 2D measurement using electronic calipers), and distribution of residual solid stool in colonic segments was also evaluated. In segments with different stool size, only the largest stool size was considered. The number of stool balls ≥6 mm was counted for each patient [11].

Grading of residual fluid was based on the maximum AP diameter of the colonic lumen submerged. For each colonic segment, scores were as follows: 1: no fluid, 2: <25% AP diameter, 3: 25 to 50% AP diameter, and 4: >50% AP diameter. In segments with different fluid levels, only the largest fluid level was considered [10].

Residual tagged solid stool scores were assigned as follows: 1: all residual solid stools untagged, 2: 1 to <25% tagged, 3: 25 to <50% tagged, 4: 50 to <75% tagged, and 5: 75 to 100% tagged [10].

The tagged appearance of residual fluid was assessed on a visual basis: tagged or nontagged [11].

### 2.5. Patient Acceptance

To evaluate patient compliance of the preparation, 101 patients answered a questionnaire in the morning before CTC. Patients were asked about global discomfort and side effects (headache, nausea, vomiting, abdominal cramps, and diarrhea) of the limited bowel preparation. Global discomfort was rated as mild, moderate, or severe.

### 2.6. Statistical Analysis

Software (SPSS version 17 for Windows) was used for statistical analyses. Residual solid stool, residual fluid, and tagging quality were compared between two groups by chi square Pearson test. Patient discomfort was compared using chi square Pearson test. *P* < 0.05 indicated a significant difference.

## 3. Results

We divided patients into two groups equally according to their CTC indications.

### 3.1. Bowel Preparation

#### 3.1.1. Residual Solid Stool

Six hundred and four bowel segments were evaluated in both supine and prone positions. One patient had a history of right colectomy.

The distribution of residual solid stool per colonic segments is presented in Table 1. Out of 604 segments, residual solid stool was noted in 409 (67%) segments. The residual solid stool ≥6 mm was noted in 226 (37%) segments, while residual solid stool <6 mm was noted in 183 (30%) segments. The number of stool balls ≥6 mm was 488 in 226 colonic segments. On average, there were 4.8 stool balls ≥6 mm, per patient. In 4-day diet group, residual solid stool ≥6 mm was noted in 41.4% (112/270) segments and <6 mm was noted in 28.8% (78/270) segments. In 2-day diet group, residual solid stool ≥6 mm was noted in 34.1% (114/334) segments and <6 mm was noted in 31.4% (105/334) segments. There was no significant difference for distribution of residual solid stool per colonic segments between 4-day and 2-day diet groups (*P* > 0.05 for all segments, Table 1).

Independent from dietregimens, out of 604 segments, residual solid stool ≥6 mm was noted in 140 (46.5%) segments in the right colon and in 86 (28.3%) segments in the left colon. That showed us that the left colon was significantly better prepared than the right colon for residual solid stool (*P* < 0.01) (Table 2).

#### 3.1.2. Residual Fluid

The distribution of residual fluid per colonic segments is presented in Table 3. In 4-day diet group residual fluid was detected in 24.8% (67/270) segments. The percentage of segments assigned a score of 1, 2, 3, or 4 for residual fluid was 75% (203/270), 21% (56/270), 3% (8/270), and 1% (3/270), respectively. In 2-day diet group, residual fluid was noted in 32.6% (109/334) segments. The percentage of segments assigned a score of 1, 2, 3, or 4 for residual fluid was 68% (227/334), 24% (81/334), 5.5% (19/334), and 2.5% (9/334). There was no significant difference for distribution of residual fluid per colonic segments between 4-day and 2-day diet group (*P* > 0.05) (Table 3).

Regardless of the dietregimens, residual fluid was detected in 29.1% (176/604) segments and the left colon was significantly better prepared than the right colon for residual fluid (*P* < 0.01) (Table 2).

#### 3.1.3. Tagging Quality

**Figure 1:**
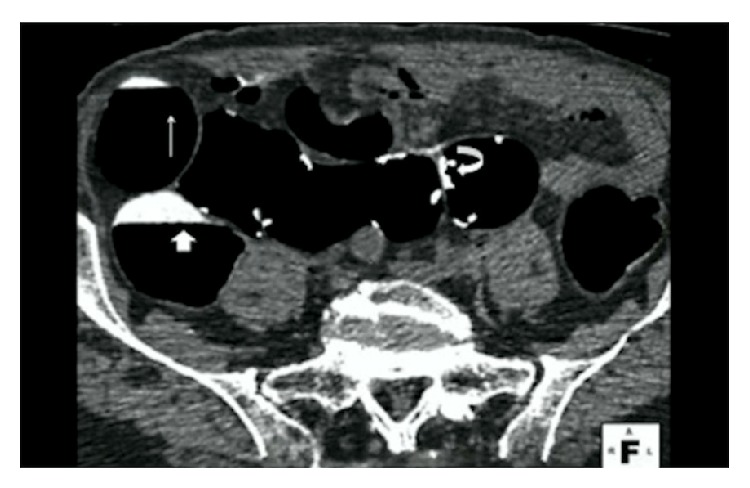
A 58-year-old man who applied 2-day dietary regimen. CT image in prone position shows tagged fluid (thin arrow) covering less than 25% of colonic lumen assigned to score 1 and 25–50% (thick arrow) assigned to score 2 for residual fluid in ascending colon. Some tagged feces (thin curved arrow) assigned to tag score 5 for residual solid stool, seen in sigmoid colon.

**Figure 2:**
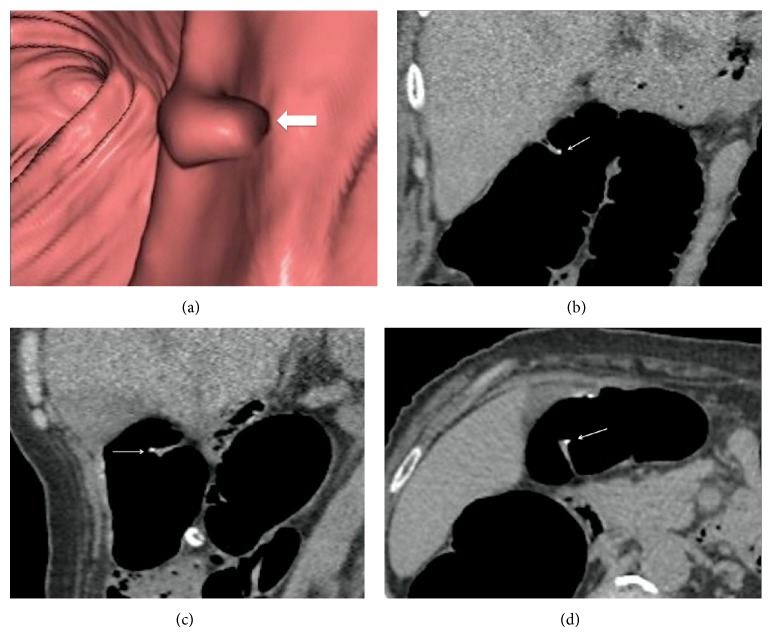
A 62-year-old woman who applied 4-day dietary regimen. 3D endoluminal CT image (a) in supine position shows polypoid structure on haustra of transvers colon (thick arrow) which coronal (b), sagittal (c), and axial (d) CT images confirmed that it was a successfully tagged (score 5) residual solid stool (thin arrow).

Tagging percentage of residual solid stool in colonic segments is presented in Table 4. Overall, the tagging quality in this study was good. In 4-day diet group, the percentage of tagging residual solid stool assigned scores of 1 (nontagged) and 5 (100% tagged) were 8.3% and 73.8%, respectively. In 2-day diet group the percentage of tagging residual solid stool assigned a score of 1 was 6.4% and score 5 was 68.3%. The tagging percentage of residual solid stool in colonic segments between 4-day and 2-day diet groups (*P* > 0.05) was not statistically significant (Table 4). The fecal tagging efficacy for ≥6 mm residual stool balls was 91.5% (Figures 1 and 2).

In 4-day diet group, nontagged fluid was detected in 7.5% (5/67) segments. In 2-day diet group nontagged fluid was detected in 8.2% (9/109) segments. There was no significant difference for residual tagged fluid between 4-day and 2-day diet group (*P* > 0.05). None of the nontagged fluid segments covered more than 50% of the colonic segments. Overall, the tagging percentage of residual fluid in colonic segments was 91% (160/176) (Figure 1).

### 3.2. Patient Acceptance

Thirty-two out of the 101 patients (31%) had complaints of the following symptoms: diarrhea (*n* = 20, 19%), abdominal cramps (*n* = 5, 5%), headache (*n* = 5, 5%), and nausea (*n* = 2, 2%). No major complication was observed during CTC examination.

**Figure 3:**
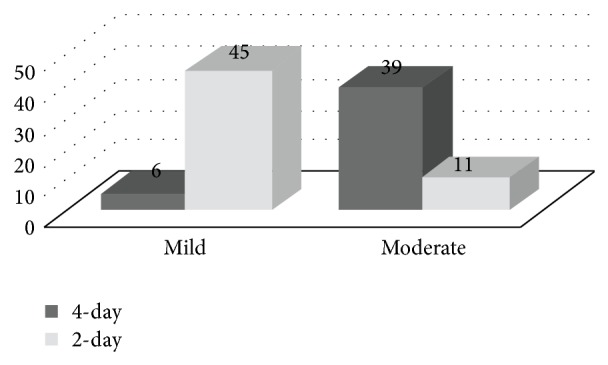
Limited bowel preparation acceptance between 4-day and 2-day diet group. Global discomfort of 2-day diet group patients was significantly higher than 4-day diet group (*P* < 0.001). None of the patients in the study stated severe discomfort.

In 4-day diet group, 6 (13%) patients reported mild discomfort and 39 (87%) patients reported moderate discomfort whereas, in 2-day diet group, 45 (80%) patients reported mild discomfort and 11 (20%) patients reported moderate discomfort. The prevalence of moderate discomfort was statistically higher in 4-day diet group as compared to patients in 2-day diet group (*P* < 0.001, Figure 3). None of the patients in the study stated severe global discomfort.

## 4. Discussion

In this study, we found that limited bowel preparation using 4-day or 2-day diet did not have significant impact on residual solid stool and residual fluid. We also found that tagging quality of the residual stool and fluid was comparable between two diet groups. However patients in 2-day diet group had better acceptance than 4-day diet group.

A study by Gollub and Flaherty [5] reported redundant/tortuous colon, patient intolerance, and angulation of colonic loops as the most common causes for incomplete CC, while a more recent study by Neerincx et al. [14] reported looping of the scope, patient discomfort, and obstructing tumor as the most frequent causes. In accordance with aforementioned studies, patient discomfort was also a common reason for incomplete CC in our study. However, poor preparation-residual colonic content during CC examination was the most common reason in our study and these patients were evaluated using CTC.

In this study, we used markedly reduced cathartic regimen with bisacodyl and senna for bowel preparation. The residual solid stool detected by CTC was higher than previous studies that used higher doses of cathartic than our study [10, 11]. We also detected lower amount of residual fluid than those studies. Higher amount of residual solid stool was an expected outcome given the markedly reduced cathartic regimen; however we unexpectedly found lower residual fluid. On the other hand, less residual fluid is generally preferable for CTC data interpretation [15, 16] since it results in shorter interpretation times [16]. Furthermore, in accordance with previous studies [10, 17] we found that the left colon was significantly better prepared than the right colon independent of the dietregimens.

The second European Society of Gastrointestinal and Abdominal Radiology (ESGAR) consensus statement on CTC agreed that fecal tagging should be used routinely for CTC [18]. The ability to tag residual colonic material at CTC allows a less burden bowel preparation for patients, compared with CC bowel preparation. Radiologists are able to distinguish high-density stool and fluid from the colonic wall in FT-CTC. Low fiber diet reduces residual bowel content and causes the homogeneous tagged solid stool [9]. In our study, fecal tagging using 2-day versus 4-day low fiber dietary with reduced cathartic bowel preparation regimens for FT-CTC did not show significant difference.

In limited bowel preparations, adequate tagging of residual contents becomes increasingly important since more fecal residual is expected. We used barium sulphate suspension as a tagging agent, which is well tolerated and carries no risk of allergic reactions [10, 11, 15]. The first ESGAR consensus statement on CTC suggested that polyps less than 5 mm should not be reported in asymptomatic screening examinations. For the symptomatic patients, reasonable minimum size for polyps to be reported is 5 or 6 mm [19]. Therefore, we assessed the tagging quality for residual stool balls ≥6 mm and found the tagging efficacy 91.5% comparable with previous studies [10, 11]. There was no significant difference between 4-day and 2-day diet groups in the tagging percentage of residual solid stool. In addition, the tagging of residual fluid was also not significant between two groups. Nontagged fluid was mostly negligible. None of the nontagged fluid segments covered more than 50% of the colonic segments.

There were several limitations of this study. First, we could not evaluate our FT technique's sensitivity and specificity for detection of polyps since our patient group was the incomplete CC patient group that was not able to undergo gold standard CC to enable the comparison. Second, the patient compliance was found to be higher. This could be partly attributed to the fact that our patients were referred to CTC, because they already had a bad experience during CC which was incomplete. Third, manual room air insufflation was used in our study. Many authors now advocate carbon dioxide administered via an automated pump to achieve colonic distention and also to allow faster absorption to reduce patient discomfort after CTC [20]. However, Shinners et al. [20] determined that there was no significant overall colonic distention advantage with either gas. Finally, regarding our questionnaire, we did not repeat it 2 or 4 weeks following CTC.

## 5. Conclusion

There was no significant difference between 2-day and 4-day limited diet groups with respect to residual solid stool, residual fluid, and tagging quality observed in FT-CTC. Patient compliance was better in 2-day limited diet group. Our study shows that 2-day limited bowel preparation regimen for fecal tag CT colonography is a safe and reasonable technique to evaluate the entire colon, particularly in incomplete conventional colonoscopy patients.

## Figures and Tables

**Table 1:** Distribution of residual solid stool in colonic segments.

Residual feces	4-day		2-day		Total		*P*
	*N*	%	*N*	%	*N*	%	
Caecum							
Absent	5	11.1%	14	25.5%	19	19.0%	0.187^*^
<6 mm	9	20.0%	10	18.2%	19	19.0%	
≥6 mm	31	68.9%	31	56.4%	62	62.0%	
Ascending colon							
Absent	9	20.0%	20	36.4%	29	29.0%	0.198^*^
<6 mm	19	42.2%	19	34.5%	38	38.0%	
≥6 mm	17	37.8%	16	29.1%	33	33.0%	
Transverse colon							
Absent	11	24.4%	19	33.9%	30	29.7%	0.279^*^
<6 mm	10	22.2%	16	28.6%	26	25.7%	
≥6 mm	24	53.3%	21	37.5%	45	44.6%	
Descending colon							
Absent	16	35.6%	21	37.5%	37	36.6%	0.605^*^
<6 mm	13	28.9%	20	35.7%	33	32.7%	
≥6 mm	16	35.6%	15	26.8%	31	30.7%	
Sigmoid colon							
Absent	19	42.2%	19	33.9%	38	37.6%	0.630^*^
<6 mm	14	31.1%	22	39.3%	36	35.6%	
≥6 mm	12	26.7%	15	26.8%	27	26.7%	
Rectum							
Absent	20	44.4%	22	39.3%	42	41.6%	0.870^*^
<6 mm	13	28.9%	18	32.1%	31	30.7%	
≥6 mm	12	26.7%	16	28.6%	28	27.7%	

^*^There was no significant difference for distribution of residual solid stool per colonic segments between 4-day and 2-day diet group (*P* > 0.05).

**Table 2:** Comparison of residual solid stool and residual fluid in the colonic segments between right and left colon independent of the dietary regimens.

	Right colon^*^		Left colon^*^		
	*N*	%	*N*	%	
Residual solid stool					
<6 mm	83	27.5%	100	33.3%	0.001^**^
≥6 mm	140	46.5%	86	28.3%	
Residual fluid 1, 2, 3, 4					
(0%, <25, 25–50, 50<)					
1	187	62.1%	241	79.5%	0.001^**^
2	89	29.6%	48	15.8%	
3	16	5.3%	11	3.6%	
4	9	3.0%	3	1.0%	

^*^Right colon: caecum, ascending colon, and transverse colon; left colon: descending colon, sigmoid colon, and rectum.

^**^The left colon was significantly better prepared than the right colon for residual solid stool and residual fluid, independent of the diet regimens (*P* < 0.01).

**Table 3:** Distribution of residual fluid in colonic segments.

Residual fluid 1, 2, 3, 4	4-day		2-day		*P*
(0%, <25, 25–50, 50<)	*N*	%	*N*	%	
Caecum					
1	24	53.3%	24	43.6%	0.199^*^
2	18	40.0%	23	41.8%	
3	3	6.7%	3	5.5%	
4	0	0.0%	5	9.1%	
Ascending colon					
1	29	64.4%	34	61.8%	0.160^*^
2	15	33.3%	13	23.6%	
3	1	2.2%	6	10.9%	
4	0	0.0%	2	3.6%	
Transverse colon					
1	34	75.6%	42	75.0%	0.853^*^
2	8	17.8%	12	21.4%	
3	2	4.4%	1	1.8%	
4	1	2.2%	1	1.8%	
Descending colon					
1	39	86.7%	42	75.0%	0.072^*^
2	2	4.4%	10	17.9%	
3	2	4.4%	4	7.1%	
4	2	4.4%	0	0.0%	
Sigmoid colon					
1	38	84.4%	43	76.8%	0.334^*^
2	7	15.6%	9	16.1%	
3	0	0.0%	3	5.4%	
4	0	0.0%	1	1.8%	
Rectum					
1	39	86.7%	40	71.4%	0.131^*^
2	6	13.3%	14	25.0%	
3	0	0.0%	2	3.6%	
4	0	0.0%	0	0.0%	

^*^There was no significant difference in distribution of residual liquid per colonic segments between 4-day and 2-day diet group (*P* > 0.05).

**Table 4:** Tagging percentage of residual solid stool in colonic segments.

Tagging percentage (1, 2, 3, 4, 5)	4-day		2-day		*P*
(0%, <25, 25–50, 50–75, 75<)	*n*	%	*n*	%	
Caecum					
Absent	5	11.1%	14	25.5%	0.115^*^
1	3	6.7%	0	0.0%	
2	3	6.7%	1	1.8%	
3	3	6.7%	2	3.6%	
4	11	24.4%	17	30.9%	
5	20	44.4%	21	38.2%	
Ascending colon					
Absent	9	20.0%	20	36.4%	0.241^*^
1	2	4.4%	0	0.0%	
2	1	2.2%	0	0.0%	
3	2	4.4%	1	1.8%	
4	5	11.1%	5	9.1%	
5	26	57.8%	29	52.7%	
Transverse colon					
Absent	10	22.2%	19	33.9%	0.423^*^
1	3	6.7%	2	3.6%	
2	1	2.2%	0	0.0%	
3	0	0.0%	1	1.8%	
4	4	8.9%	2	3.6%	
5	27	60.0%	32	57.1%	
Descending colon					
Absent	16	35.6%	21	37.5%	0.603^*^
1	2	4.4%	3	5.3%	
2	0	0.0%	2	3.7%	
3	2	4.4%	1	1.8%	
4	2	4.4%	4	7.1%	
5	23	51.2%	25	44.6%	
Sigmoid colon					
Absent	19	42.2%	19	33.9%	0.172^*^
1	4	8.9%	6	10.7%	
2	0	0.0%	3	5.4%	
3	2	4.4%	1	1.8%	
4	0	0.0%	5	8.9%	
5	20	44.4%	22	39.3%	
Rectum					
Absent	20	44.4%	23	41.1%	0.499^*^
1	1	2.2%	3	5.4%	
2	0	0.0%	1	1.8%	
3	0	0.0%	1	1.8%	
4	3	6.7%	8	14.3%	
5	21	46.7%	20	35.7%	

^*^There was no significant difference for tagging percentage of residual solid stool in colonic segments between 4-day and 2-day diet groups (*P* > 0.05).
